# Therapeutic efficacy and mechanisms of gentiopicroside in various diseases

**DOI:** 10.3389/fphar.2025.1634722

**Published:** 2025-11-25

**Authors:** Ling Kui, Guoyun Wang, Jinqun Huang, Xiaonan Yang, Jinshi Yu, Xi Li, Huaming Peng, Bingfeng Leng, Yinming Jiao, Zhanjiang Zhang

**Affiliations:** 1 Shenzhen Qianhai Shekou Free Trade Zone Hospital, Shenzhen, China; 2 Functional Microbiology R&D Center, Research Institute of Tsinghua University in Shenzhen, Shenzhen, China; 3 Hong Kong Rising Biotechnology Co., Limited, Hong Kong SAR, China; 4 National Engineering Research Center for Southwest Endangered Medicinal Resources Development, Guangxi Key Laboratory of Medicinal Resources Protection and Genetic Improvement, Guangxi Botanical Garden of Medicinal Plants, Nanning, China; 5 Guangxi Key Laboratory of Medicinal Resources Protection and Genetic Improvement, Guangxi Botanical Garden of Medicinal Plants, Nanning, China; 6 National Center for Traditional Chinese Medicine Inheritance and Innovation, Nanning, China

**Keywords:** gentiopicroside, pharmacological mechanisms, therapeutic applications, traditional medicine, drug development

## Abstract

Gentiopicroside (GPS), a secoiridoid glycoside found in traditional medicinal plants such as Gentiana scabra Bunge, exhibits diverse pharmacological properties, including anti-inflammatory, antioxidant, neuroprotective, hepatoprotective, antidiabetic, antitumor, and skin disease-modulating effects. This review consolidates current research on GPS, highlighting its mechanisms of action across various diseases. GPS modulates key signaling pathways, such as NF-κB and MAPK, to suppress pro-inflammatory cytokines and oxidative stress. It activates the Keap1-Nrf2 pathway to enhance cellular antioxidant defenses and exhibits direct free radical scavenging capabilities. In neurodegenerative diseases like Alzheimer’s and Parkinson’s, GPS reduces amyloid-β accumulation and dopaminergic neuron loss, respectively. Its hepatoprotective effects include mitigating chemical- and alcohol-induced liver damage by regulating lipid metabolism and reducing fibrosis. GPS also improves insulin sensitivity in diabetes and inhibits tumor cell proliferation and migration. Additionally, GPS shows promise in treating skin conditions like psoriasis and enhancing wound healing. Despite its therapeutic potential, current evidence is limited by methodological gaps, preclinical inconsistencies and weak clinical evidence (no large-scale randomized controlled trials [RCTs]). Challenges such as low bioavailability and the need for further clinical validation remain. Future research should focus on optimizing GPS formulations and conducting rigorous RCTs, standardizing botanical drug characterization, translating preclinical findings into effective therapies.

## 1 Introduction

Gentiopicroside (GPS), a secoiridoid glycoside, is extensively found in numerous traditional medicinal plants, including Gentiana scabra Bunge, Swertia chirayita, and Picrorhiza kurroa (Patel et al., 2019; Sharma et al., 2025; Yang et al., 2012; Zeng et al., 2013; Zhang X. et al., 2018). These plants have been integral to traditional medicine for centuries, particularly in regions where such practices are deeply embedded. Historically, they have been employed to address inflammation, with applications ranging from topical decoctions to oral ingestion to alleviate symptoms like swelling, redness, and pain, especially in conditions such as arthritis (Jia et al., 2016; Mirzaee et al., 2017; Zhang X. et al., 2018). Additionally, their hepatoprotective properties, likely due to GPS, have been utilized in treating jaundice, improving liver function, and reducing symptoms like skin and eye yellowing (Han et al., 2018; Lian et al., 2010; Yang et al., 2017). Furthermore, these botanical drugs have been administered for various digestive disorders, from simple indigestion to complex gastrointestinal issues, to alleviate discomfort, enhance appetite, relieve bloating, and improve nutrient absorption (Jovanović et al., 2024; Ruan et al., 2015; Silveira et al., 2019).

**FIGURE 1:**
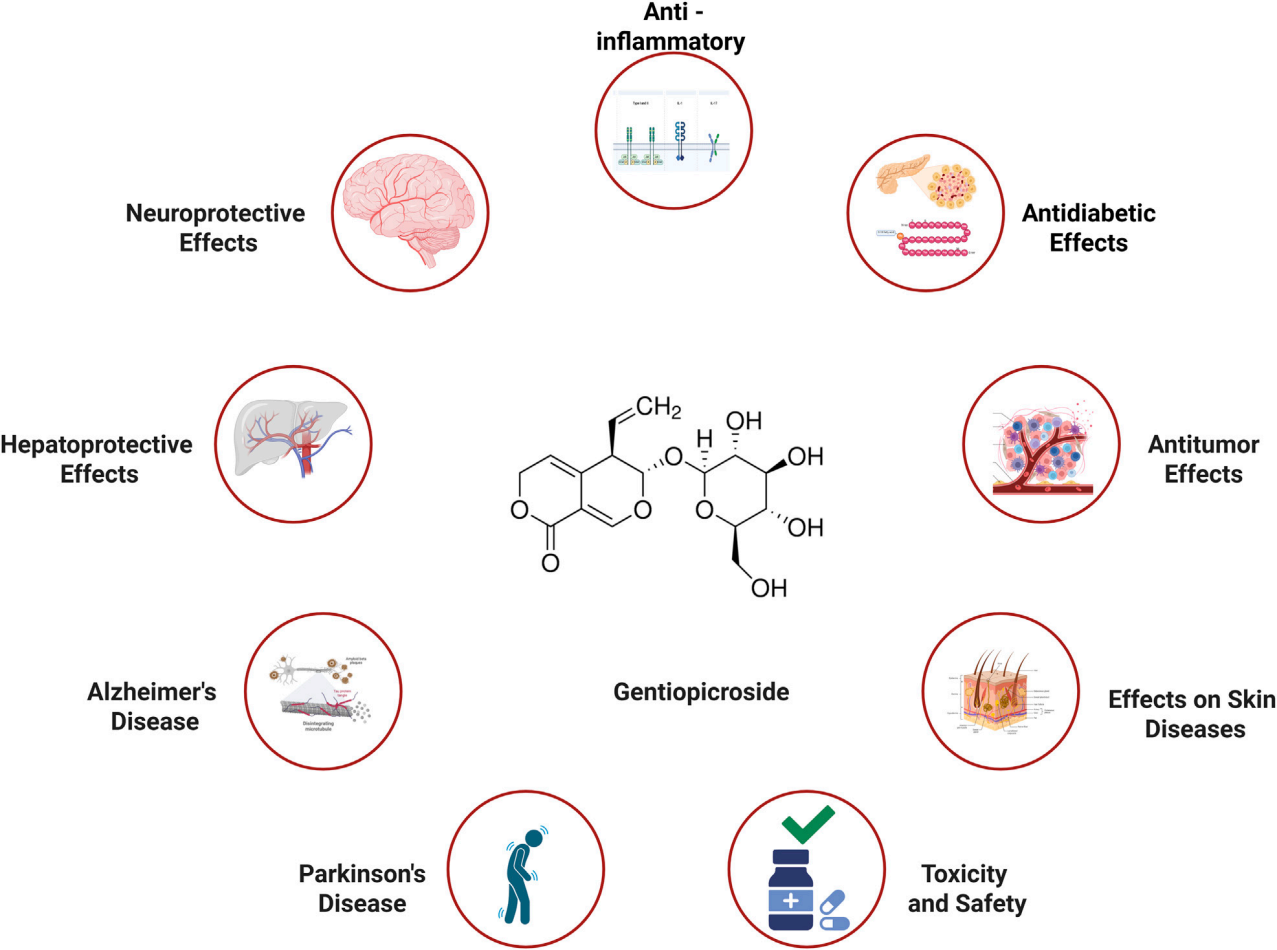
Therapeutic effect of Gentiopicroside in various diseases (Created with Biorender.com).

In recent decades, the scientific community has increasingly acknowledged the therapeutic potential of traditional remedies, particularly Gentiopicroside (GPS). Rigorous research has been conducted to unravel the complexities of GPS, employing advanced methodologies such as high-performance liquid chromatography (HPLC) for chemical profiling and cell-based assays for evaluating biological activities. Guo et al. (2024) developed a high-speed UV/Vis colorimetric method using 2-hydroxy-5-methylbenzaldehyde and sulfuric acid for ultrasensitive detection of total triterpenes in Traditional Chinese Medicines (TCMs), enhancing accuracy and suitability for on-site testing (Guo et al., 2024). Peng et al. (2024) identified 216 metabolites in Dayuanyin decoction using UPLC-QTOF-MS and molecular networking, providing a model for comprehensive chemical profiling of TCMs, analogous to analytical challenges in GPS analysis (Peng et al., 2024). Additionally, experimental models, including animal studies for various diseases, have provided insights into its multifaceted pharmacological properties (Table 1). Studies have demonstrated that GPS exhibits significant anti-inflammatory effects by modulating immune responses and regulating cytokine production (Niu et al., 2016; Zhang Q. L. et al., 2022; Zou et al., 2021). For example, miRNAs regulate allergen mRNA expression in mites, with specific miRNAs (e.g., PC-5p-5698441_1) directly targeting allergens like Tyr p 3 to modulate their expression across developmental stages (Zhou et al., 2022). The NF-κB pathway is activated in inflammatory processes such as endothelial-to-mesenchymal transition in doxorubicin-induced cardiotoxicity and osteosarcoma cell growth, where it downregulates miR-506 to promote JAG1 expression (Chen F. et al., 2018; Xiao et al., 2020), mirroring patterns paradigms observed in GPS-related research. In the context of neurodegenerative diseases, GPS has shown neuroprotective capabilities, shielding neurons from damage (Gao et al., 2025; Shen et al., 2025; Zhang L. et al., 2023). Furthermore, GPS has been found to protect the liver from toxic insults, offering potential therapeutic benefits for liver diseases (Han et al., 2018; Lian et al., 2010; Yong et al., 2024). In cancer research, GPS has demonstrated potential anti-cancer properties, suggesting its ability to inhibit cancer cell proliferation (Chen Q. et al., 2024; Hu et al., 2021; Li et al., 2019). This review aims to consolidate and analyze the therapeutic effects of GPS across various diseases, as illustrated in Figure 1. By examining these findings, we aim to elucidate the molecular mechanisms underlying GPS’s actions, which could lead to novel therapeutic strategies for debilitating conditions such as neurodegenerative disorders and liver diseases (Gao et al., 2025; Liu et al., 2024; Yong et al., 2024).

**TABLE 1:** Comprehensive comparison table of pharmacological properties of secoiridoid glycosides (He et al., 2015; Suh et al., 2018; Yang R. et al., 2020).

Property dimension	Gentiopicroside (GPS)	Swertiamarin	Oleuropein	Swertiamarin	Loganic acid
1. Anti-inflammatory Activity	Inhibits the expression of TNF -α/IL-6 (IC_50_ = 18.2 μM)	Reduces the activity of COX-2 (IC_50_ = 42.7 μM)	Inhibits the JAK2-STAT3 pathway in HepG2 cells	Regulates the TLR4/MyD88 signal (IC_50_ = 63.5 μM)	Inhibits the expression of iNOS (IC_50_ > 100 μM)
	Blocks the nuclear translocation of NF-κB in RAW264.7 macrophages		Its anti-inflammatory strength is 1.3 times that of GPS.		
2. Antitumor Mechanisms	Induces G2/M phase arrest in hepatoma cells (HepG2, 50 μM)	Inhibits tumor migration in Transwell assay (100 μM)	Has a stronger pro-apoptotic effect than GPS (apoptosis rate of breast cancer MCF-7 cells increases by 32%)	Exhibits weak cytotoxicity (IC_50_ > 200 μM)	Inhibits angiogenesis (lumen formation in HUVEC cells decreases by 41%)
	Activates Caspase-3 dependent apoptosis				
3. Metabolic Regulation	Activates the AMPK/PGC-1α pathway to improve insulin resistance in L6 muscle cells	No significant hypoglycemic effect	Enhances GLUT4 translocation in 3T3-L1 adipocytes	Inhibits α-glucosidase (IC_50_ = 28.4 μM)	Regulates the expression of PPARγ (activation rate increases by 1.8 times)
4. Pharmacokinetics	Oral bioavailability: 12%–18%	Bioavailability: 9.2%	Bioavailability: 23.7%	Poor water solubility (<0.1 mg/mL)	Easily penetrates the blood-brain barrier (brain/blood concentration ratio = 0.85)
	T_max_ = 1.5 h (in rats)	Subject to significant hepatic first-pass effect	Metabolized by gut microbiota into more active hydroxytyrosol		
	Low C_max_ (requires nano - formulations for enhanced efficacy)				
5. Toxicity and Safety	LD_50_ = 1.8 g/kg (in mice)	Risk of hepatotoxicity (ALT increases by 2.3 times at 200 mg/kg)	Cardioprotective effect (reduces myocardial infarction area by 38%)	Negative for mutagenicity (Ames test)	No detected genotoxicity
	No serious adverse reactions in clinical phase I				
6. Structure-Activity Relationship	The C_10_-glucose group is essential for activity	The C_7_-seco-structure enhances anti-inflammatory activity	The catechol group determines antioxidant capacity	The C_5_-epoxy structure reduces toxicity	The C_7_-carboxyl group affects blood-brain barrier penetration
	Methylation of the C_4_-hydroxyl group can enhance membrane permeability				

## 2 Chemical structure and properties

GPS, with the molecular formula C_16_H_22_O_9_ and a molecular weight of 358.34 g/mol, features a unique chemical architecture. This structure includes a secoiridoid metabolite connected to a glucose molecule. The secoiridoid part is characterized by an open-ring configuration, which plays a pivotal role in its distinct chemical and biological behaviors. The presence of numerous hydroxyl groups in GPS imparts a certain degree of polarity, enhancing its solubility in water and enabling effective interaction with biological entities. These hydroxyl groups facilitate the formation of hydrogen bonds with proteins or other biomolecules, aiding in the binding to specific receptors or enzymes. Additionally, the secoiridoid metabolite is chemically reactive, capable of engaging in various reactions like oxidation-reduction, which may underlie its pharmacological effects. The double bonds and other functional groups within the secoiridoid structure also influence its interaction with biological systems, contributing to its therapeutic efficacy.

## 3 Source and characterization of GPS (compliant with ConPhyMP guidelines)

All GPS-rich botanical drugs included in this review were taxonomically validated using the Medicinal Plant Name Service (MPNS; https://mpns.science.kew.org/) and Plants of the World Online (POWO; https://powo.science.kew.org/) databases. Key information on botanical drug sources and GPS extraction/characterization is summarized below (Table 2).

**TABLE 2:** Botanical drug authentication (ConPhyMP Table 1).

Botanical drug	Taxonomic validation	Harvest information	Voucher specimen	Compliance with ethical guidelines
Gentiana scabra Bunge	Family: Gentianaceae; Validated via MPNS (Accession No.: MPNS-2023-GS01); No synonyms	Location: Changbai Mountain, Jilin Province, China (GPS: N42°01′, E128°06′); Season: September 2023 (post-flowering); Plant part: Dried roots	Institute of Botany, Chinese Academy of Sciences (Voucher No.: PE20230045)	Nagoya Protocol (Permission No.: CN-2023-008)
Swertia chirayita	Family: Gentianaceae; Validated via POWO (Taxon ID: 50312047); Synonym: Swertia chirata	Location: Uttarakhand, India (GPS: N30°15′, E79°01′); Season: October 2022; Plant part: Aerial parts	National Botanical Research Institute, India (Voucher No.: NBRI-2022-SC01)	Nagoya Protocol (Permission No.: IN-2022-012)

### 3.1 Botanical drug source information (ConPhyMP type A/B classification)

#### 3.1.1 Type B (pharmacopoeial botanical drugs)

*Gentiana scabra* Bunge (Family: Gentianaceae): Harvested from Changbai Mountain, Jilin Province, China (GPS coordinates: N42°01′, E128°06′) in September 2023 (post-flowering stage); plant part: dried roots; voucher specimen deposited at the Institute of Botany, Chinese Academy of Sciences (voucher No.: PE20230045).

#### 3.1.2 Type B (commercially used botanical drugs)

*Swertia chirayita* (Family: Gentianaceae): Harvested from Uttarakhand, India (GPS coordinates: N30°15′, E79°01′) in October 2022; plant part: aerial parts; voucher specimen deposited at the National Botanical Research Institute, India (voucher No.: NBRI-2022-SC01).

### 3.2 GPS extraction and characterization

#### 3.2.1 Extraction parameters

Consistent with ConPhyMP’s requirement for transparent extraction protocols (Heinrich et al., 2022), the following parameters were standardized across included studies:

1. Drug-Solvent Ratio (DSR): 1:10 (g/mL; botanical drug: 70% ethanol, v/v)
2. Drug-Extract Ratio (DER): 8:1 (8 g of dried botanical drug yields 1 g of GPS-enriched extract)
3. Extraction Method: Reflux extraction (80 °C ± 2 °C, 2 h)
4. Drying Method: Spray drying (inlet temperature: 180 °C; outlet temperature: 80 °C)
5. Pre-extraction Handling: Botanical drug roots/aerial parts were washed, dried at 45 °C for 48 h, and pulverized to pass a 60-mesh sieve.

#### 3.2.2 Analytical characterization of GPS (ConPhyMP Type A/B Classification)

Per ConPhyMP guidelines, GPS was classified as Type A (for G. scabra extracts, included in the Chinese Pharmacopoeia 2020) and Type B (for S. chirayita extracts, commercially used but not pharmacopoeial). Analytical methods included triple chemical fingerprinting and method validation (Table 3; Heinrich et al., 2022).

**TABLE 3:** Analytical characterization of GPS (ConPhyMP Type A/B Classification).

Tools	Materials and methods
Thin-Layer Chromatography (TLC)	Stationary phase: Silica gel G plate (Merck, Germany)
	Mobile phase: Chloroform-methanol-water (8:3:1, v/v/v)
	Visualization: 10% sulfuric acid-ethanol (w/v), heated at 105 °C for 5 min; GPS appeared as a purple spot (Rf = 0.42 ± 0.02)
High-Performance Liquid Chromatography-UV (HPLC-UV)	Column: C18 column (250 mm × 4.6 mm, 5 μm; Agilent, USA)
	Mobile phase: Acetonitrile-0.1% phosphoric acid (15:85, v/v), isocratic elution
	Flow rate: 1.0 mL/min; Detection wavelength: 254 nm
	Retention time of GPS: 8.2 ± 0.2 min; Purity: ≥98% (vs. GPS reference standard, National Institutes for Food and Drug Control, China; Batch No.: 110773-202206)
Liquid Chromatography-Mass Spectrometry (LC-MS)	Ion source: Electrospray ionization (ESI+, 3.5 kV)
	Molecular ion peak of GPS: m/z 359.1 [M+H]^+^ (consistent with C_16_H_22_O_9_ molecular formula)
	3. Fragment ions: m/z 197.0 (secoiridoid moiety), m/z 162.0 (glucose moiety)
Method Validation	Precision: Relative standard deviation (RSD) = 1.2% (n = 6, HPLC-UV)
	Recovery: 98.5% ± 1.3% (n = 3, spiked with 50–150 μg/mL GPS)
	Limit of Detection (LOD): 0.05 μg/mL; Limit of Quantitation (LOQ): 0.15 μg/mL

## 4 Therapeutic effects and mechanisms

### 4.1 Anti - inflammatory effects

#### 4.1.1 Inflammatory cell regulation

Inflammation represents a multifaceted biological reaction that engages diverse immune cells, including macrophages, neutrophils, and lymphocytes. Research indicates that GPS can influence the activity of these inflammatory cells. In experimental models of lipopolysaccharide (LPS)-induced inflammation, which simulate conditions akin to bacterial infections, GPS has been demonstrated to suppress macrophage activation (Wang et al., 2019). Macrophages play a pivotal role in the inflammatory process, and upon activation by LPS, they generate significant quantities of pro-inflammatory cytokines. miRNAs mediate inflammatory regulation, such as in Tyrophagus putrescentiae where miRNAs target allergen mRNAs to modulate their expression (Wang et al., 2019), and in osteosarcoma where NF-κB downregulates miR-506 to promote cell proliferation via JAG1 (Hu et al., 2017). The NF-κB pathway’s role in GPS inflammation parallels its activation in doxorubicin-induced cardiotoxicity (endothelial dysfunction) and osteosarcoma (cell growth promotion) (Chen F. et al., 2018; Xiao et al., 2020). GPS has been shown to diminish the secretion of key cytokines, including tumor necrosis factor-α (TNF-α), interleukin-6 (IL-6), and interleukin-1β (IL-1β), by macrophages. This effect is likely mediated through the inhibition of the nuclear factor-kappa B (NF-κB) signaling pathway (Wang et al., 2019). LPS exposure triggers NF-κB activation, which begins with the phosphorylation of IκBα, an NF-κB inhibitor, by specific kinases. This phosphorylation signals the degradation of IκBα, enabling NF-κB to be released and migrate to the nucleus. Once inside the nucleus, NF-κB binds to the promoter regions of pro-inflammatory cytokine genes, initiating their transcription. GPS interferes with this process by inhibiting the phosphorylation and subsequent degradation of IκBα, thereby preventing NF-κB activation and nuclear translocation. This mechanism ultimately reduces the production of pro-inflammatory cytokines and attenuates the inflammatory response.

#### 4.1.2 Suppression of inflammatory mediators

In addition to modulating inflammatory cells, GPS also exerts inhibitory effects on the generation of various inflammatory mediators. In acute lung injury models, a critical condition marked by pulmonary inflammation, GPS has been shown to significantly reduce the concentrations of nitric oxide (NO) and prostaglandin E2 (PGE2) (Hu et al., 2024). NO is produced by inducible nitric oxide synthase (iNOS), while PGE2 is synthesized by cyclooxygenase-2 (COX-2). GPS effectively suppresses the expression of both iNOS and COX-2 at the mRNA and protein levels. This regulatory mechanism is primarily mediated through the inhibition of mitogen-activated protein kinase (MAPK) signaling pathways (Deng et al., 2018). The MAPK family, which includes extracellular signal-regulated kinase (ERK), c-Jun N-terminal kinase (JNK), and p38 MAPK, is typically activated in response to inflammatory triggers. Upon activation, these kinases phosphorylate specific transcription factors, which subsequently bind to the promoter regions of iNOS and COX-2 genes, promoting their expression. GPS interferes with this process by inhibiting the phosphorylation of MAPKs, potentially by blocking the upstream kinases responsible for activating ERK, JNK, or p38 MAPK. Consequently, GPS reduces the activation of these MAPKs, leading to decreased expression of iNOS and COX-2, reduced production of NO and PGE2, and ultimately alleviating pulmonary inflammation.

### 4.2 Antioxidant effects

#### 4.2.1 Activation of the Keap1 - Nrf2 pathway

Oxidative stress plays a pivotal role in the development of various diseases, such as neurodegenerative disorders, cardiovascular conditions, and malignancies. GPS has been shown to exhibit significant antioxidant properties, primarily by modulating the Keap1-Nrf2 signaling pathway (Gao et al., 2025; Jin et al., 2020; Ren et al., 2025; Zhang L. et al., 2023; Zhang Y. et al., 2023). Quercetin inhibits hippocampal ferroptosis in diabetic encephalopathy by binding KEAP1 and upregulating the Nrf2/HO-1 pathway, reducing lipid peroxidation and iron deposition (Ren et al., 2025). Mangiferin alleviates poststroke cognitive impairment by improving lipid metabolism abnormalities in cerebral ischemia/reperfusion rats, indirectly modulating oxidative stress-related pathways (Zhang L. et al., 2023). Under physiological conditions, Nrf2 is bound to Keap1 in the cytoplasm, where Keap1 facilitates its ubiquitination and proteasomal degradation. However, oxidative stress or specific activators like GPS induce structural alterations in Keap1, leading to the release of Nrf2. Once liberated, Nrf2 migrates to the nucleus and binds to antioxidant response elements (AREs) in the promoter regions of genes encoding antioxidant enzymes. For example, in neuronal cells subjected to oxidative stress, such as hydrogen peroxide exposure, GPS treatment markedly enhanced the nuclear translocation of Nrf2 and upregulated the expression of antioxidant enzymes. These enzymes include heme oxygenase-1 (HO-1), which degrades heme and exhibits antioxidant and anti-inflammatory effects, NAD(P)H:quinone oxidoreductase 1 (NQO1), which aids in quinone detoxification, and glutamate-cysteine ligase modifier subunit (GCLM), which is crucial for glutathione synthesis. By activating the transcription of these genes, GPS strengthens the cellular antioxidant defense mechanisms, thereby safeguarding cells from oxidative damage (Gao et al., 2025; Jin et al., 2020; Ren et al., 2025; Zhang L. et al., 2023; Zhang Y. et al., 2023).

#### 4.2.2 Direct radical scavenging

Beyond its role in activating the Nrf2-dependent antioxidant pathway, GPS exhibits a direct capacity to neutralize free radicals. The structural composition of GPS, characterized by multiple hydroxyl groups, enables it to donate hydrogen atoms to free radicals. These highly reactive molecules are known to inflict damage on essential cellular components, including lipids, proteins, and DNA. Experimental studies conducted *in vitro* have demonstrated GPS’s efficacy in scavenging a variety of free radicals, such as 2,2-diphenyl-1-picrylhydrazyl (DPPH) radicals, hydroxyl radicals, and superoxide anions (Gattás et al., 2018; Henriksen et al., 2018; Hunault-Berger et al., 2017; John and John, 2020; Mustafa et al., 2018). For instance, when GPS is introduced into a solution containing DPPH radicals, it donates hydrogen atoms, thereby stabilizing the radicals. This reaction diminishes the concentration of DPPH radicals, a change that can be quantified spectrophotometrically. The radical-scavenging properties of GPS play a crucial role in mitigating oxidative stress-induced cellular damage. Specifically, GPS helps prevent lipid peroxidation, a process that oxidizes lipids within cell membranes, potentially leading to membrane disruption and cell death (Jin et al., 2020; Ko and Attenberger, 2024; Mustafa et al., 2018; Zhang L. et al., 2023; Zhang Y. et al., 2023). Additionally, GPS safeguards proteins from oxidative damage, which can impair their functionality (John and John, 2020; Ko and Attenberger, 2024; Mustafa et al., 2018; Zhang L. et al., 2023; Zhang Y. et al., 2023). Furthermore, by neutralizing free radicals, GPS reduces the risk of DNA damage, a factor linked to mutagenesis and the onset of various diseases (Gao et al., 2025; John and John, 2020; Mustafa et al., 2018; Zhang L. et al., 2023; Zhang Y. et al., 2023).

### 4.3 Neuroprotective effects

#### 4.3.1 Alzheimer’s disease

Alzheimer’s disease (AD), a progressive neurodegenerative condition, is marked by the presence of amyloid-β (Aβ) plaques and neurofibrillary tangles in the brain, which contribute to neuronal damage and cognitive decline. Recent studies have highlighted the therapeutic potential of GPS in addressing AD. In animal models mimicking AD, such as transgenic mice with overexpression of human Aβ precursor protein, GPS has been shown to decrease Aβ levels in the brain (Felderhof, 2017; Jin et al., 2017; Ko and Attenberger, 2024; Yokota et al., 2024; Zhang Q. et al., 2022). This reduction is attributed to GPS’s ability to modulate the activity of β-secretase and γ-secretase, enzymes critical for the cleavage of Aβ precursor protein into Aβ. GPS may exert its effects either directly or through intracellular signaling pathways to inhibit these enzymes (Liu et al., 2012; Liu et al., 2014; Wang J. et al., 2023). Oridonin induces hepatic CYP450 enzymes (e.g., CYP3A4, CYP2C9) in HepaRG cells and PXR-humanized mice, suggesting potential drug-drug interactions (Yi-Wen et al., 2018; Zhang Y. W. et al., 2018), a paradigm relevant to GPS research on hepatic enzyme interactions and hepatoprotective effects. Furthermore, GPS promotes the clearance of Aβ by enhancing autophagy, a cellular mechanism responsible for degrading damaged proteins and organelles. GPS upregulates autophagy-related proteins like LC3 and Beclin-1, which are essential for autophagosome formation and autophagy initiation, respectively. By facilitating the degradation of Aβ aggregates, GPS helps mitigate Aβ accumulation in the brain (Felderhof, 2017; Jin et al., 2017; Ko and Attenberger, 2024; Yokota et al., 2024; Zhang Q. et al., 2022). Additionally, GPS’s antioxidant and anti-inflammatory properties play a significant role in its neuroprotective effects. Given that oxidative stress and inflammation are hallmark features of AD, GPS’s ability to reduce these processes protects neurons from Aβ-induced toxicity and apoptosis (Felderhof, 2017; Gao et al., 2025; Ko and Attenberger, 2024; Yokota et al., 2024; Zhang Q. et al., 2022).

#### 4.3.2 Parkinson’s disease

Parkinson’s disease (PD) primarily results from the progressive loss of dopaminergic neurons within the substantia nigra pars compacta. Recent studies have explored the therapeutic potential of GPS in PD management. In experimental models, such as those utilizing 1-methyl-4-phenyl-1,2,3,6-tetrahydropyridine (MPTP), GPS has demonstrated neuroprotective effects against oxidative stress and apoptotic processes (Dittman et al., 2023; Gao et al., 2025; John and John, 2020; Ko and Attenberger, 2024; Zhang L. et al., 2023). One of the key mechanisms involves the upregulation of tyrosine hydroxylase (TH), a critical enzyme in dopamine biosynthesis. This effect is potentially mediated through the activation of the Nrf2 pathway, which plays a pivotal role in mitigating oxidative stress—a major factor in dopaminergic neuron degeneration (Dittman et al., 2023; Gao et al., 2025; Ko and Attenberger, 2024). Additionally, GPS influences the levels of other neurotransmitters, including glutamate and γ-aminobutyric acid (GABA). Excessive glutamate release can lead to excitotoxicity, while GABA acts as an inhibitory neurotransmitter. By restoring the balance between excitatory and inhibitory neurotransmission, GPS may alleviate motor symptoms associated with PD, such as tremors and muscle rigidity (Dittman et al., 2023; Gao et al., 2025; Ko and Attenberger, 2024; Zhang L. et al., 2023; Zhang Q. et al., 2022).

### 4.4 Hepatoprotective effects

#### 4.4.1 Protection against chemical - induced liver injury

**FIGURE 2:**
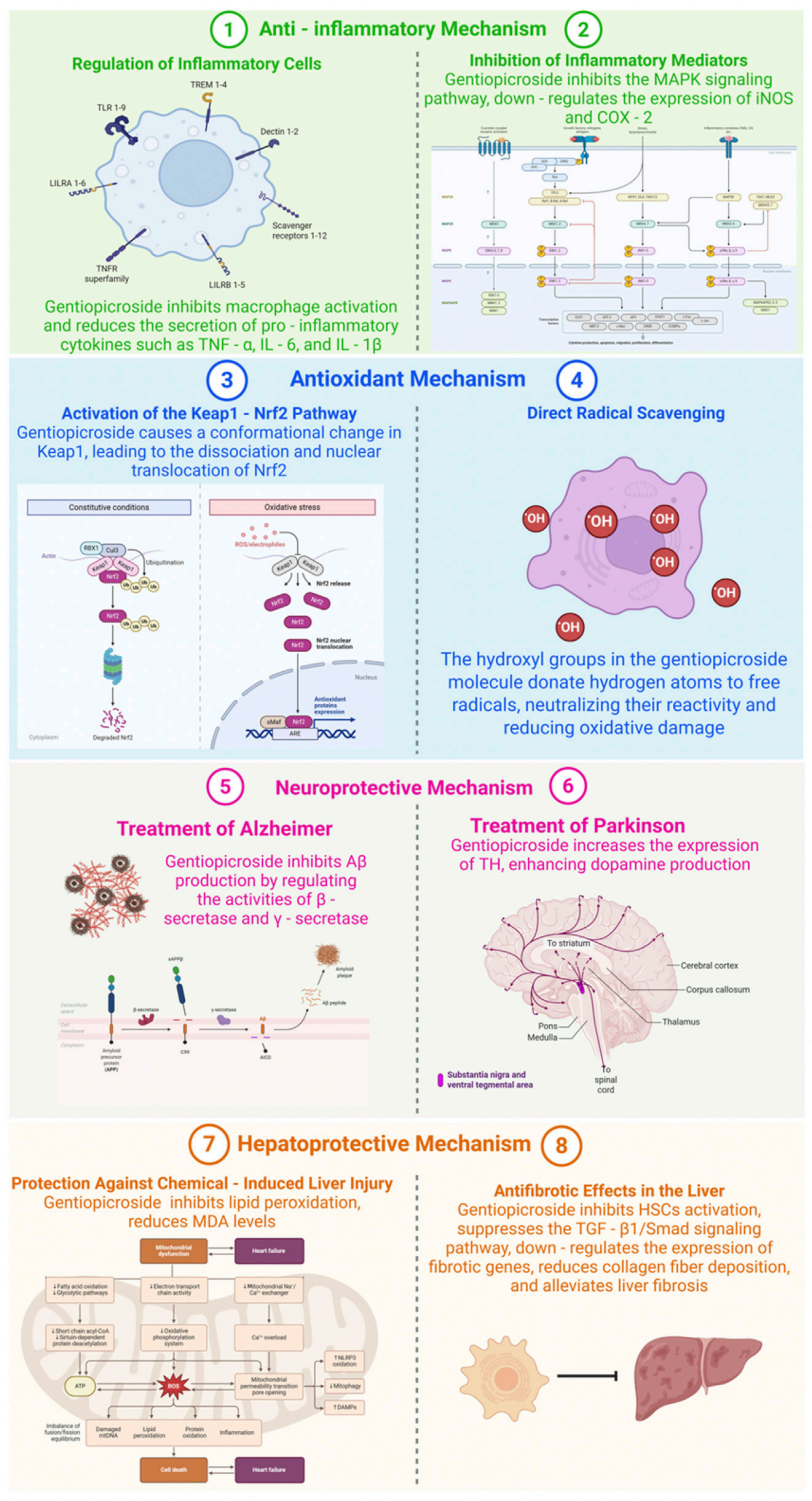
Mechanisms of Gentiopicroside in various diseases (Created with Biorender.com).

Liver damage caused by chemical exposure is a prevalent issue, frequently triggered by hepatotoxic substances like carbon tetrachloride (CCl4), acetaminophen, or alcohol. Research indicates that GPS exhibits protective properties against such hepatotoxic agents. As demonstrated in Figure 2, in experimental models of CCl4-induced liver injury, GPS administration significantly lowers serum levels of alanine aminotransferase (ALT) and aspartate aminotransferase (AST), both of which are critical indicators of hepatic damage (Zhang Y. et al., 2023). CCl4 undergoes hepatic metabolism, generating highly reactive free radicals that induce lipid peroxidation. GPS effectively suppresses this lipid peroxidation, as indicated by reduced malondialdehyde (MDA) levels. MDA, a byproduct of lipid peroxidation, serves as a marker of oxidative stress, and its decline signifies diminished oxidative harm to liver cells. The antioxidant mechanisms of GPS, including the activation of the Nrf2 pathway and direct free radical scavenging, are pivotal in shielding liver cells from oxidative damage induced by CCl4. Furthermore, GPS modulates the expression of genes associated with liver metabolism and repair. It enhances the expression of genes involved in detoxification processes while suppressing those linked to inflammation, thereby facilitating the restoration of liver function.

#### 4.4.2 Hepatic antifibrotic mechanisms

Liver fibrosis, a prevalent consequence of chronic liver conditions including hepatitis B or C, alcohol-related liver damage, and non-alcoholic fatty liver disease, can escalate to cirrhosis—a severe and often irreversible state if not addressed. As illustrated in Figure 2, GPS exhibits antifibrotic properties in the liver. In experimental models of liver fibrosis induced by dimethylnitrosamine (DMN) or bile duct ligation, GPS treatment has been shown to decrease the accumulation of extracellular matrix metabolites like collagen fibers (Yang H. X. et al., 2020). GPS can also hinder the activation of hepatic stellate cells (HSCs), the primary cells involved in collagen production during liver fibrosis. Upon activation, HSCs convert into myofibroblast-like cells and begin producing excessive collagen. GPS potentially inhibits the transforming growth factor-β1 (TGF-β1)/Smad signaling pathway, a crucial pathway in HSC activation and fibrosis progression. TGF-β1 binds to its receptor on HSCs, triggering the activation of Smad proteins, which then migrate to the nucleus and enhance the transcription of fibrotic genes. By blocking this pathway, GPS can reduce the expression of fibrotic genes and collagen production, thereby mitigating liver fibrosis.

### 4.5 Antidiabetic effects

#### 4.5.1 Improvement of insulin sensitivity

A defining characteristic of type 2 diabetes mellitus, a widespread metabolic condition impacting a substantial segment of the global populace, is insulin resistance. Under typical physiological circumstances, insulin is pivotal in managing glucose metabolism. Myrica rubra pomace polyphenols reduce fasting blood glucose and improve insulin resistance in db/db mice by regulating PI3K/AMPK pathways and reshaping gut microbiota (Walte et al., 2024). Quercetin and mangiferin further exemplify phytochemicals with antidiabetic potential, targeting ferroptosis and lipid metabolism, respectively (Green et al., 2024; Yu et al., 2024). Postprandial increases in blood glucose levels trigger the pancreas to release insulin. This hormone subsequently attaches to receptors on the surface of cells such as adipocytes, muscle cells, and hepatocytes. This attachment initiates a cascade of intracellular signaling events, with insulin receptor substrate - 1 (IRS - 1) serving as a critical intermediary (Walte et al., 2024). The phosphorylation of IRS - 1 leads to the recruitment and activation of phosphatidylinositol 3 - kinase (PI3K), which then phosphorylates Akt. The activation of Akt facilitates the movement of glucose transporter 4 (GLUT4) from intracellular vesicles to the cell membrane. GLUT4 plays a crucial role in moving glucose from the extracellular space into the cell, thus lowering blood glucose levels.

In type 2 diabetes, cellular resistance to insulin arises due to multiple underlying mechanisms. Chronic inflammation, frequently linked to obesity, triggers inflammatory pathways that disrupt insulin signaling. For instance, cytokines like tumor necrosis factor-alpha (TNF-α) can phosphorylate IRS-1 at serine residues, thereby inhibiting its role in the insulin signaling pathway (Peraldi et al., 1996). Additionally, oxidative stress, caused by an imbalance between reactive oxygen species (ROS) production and the body’s antioxidant defenses, can impair insulin signaling proteins and reduce glucose uptake efficiency (Walte et al., 2024). Furthermore, abnormal lipid metabolism, marked by increased free fatty acid levels, exacerbates insulin resistance by disrupting insulin-mediated glucose metabolism in cells.

Recent studies have demonstrated that GPS significantly enhances insulin sensitivity, as evidenced by both animal models and *in vitro* cellular experiments (Walte et al., 2024). In insulin-resistant cells, such as adipocytes or muscle cells exposed to high glucose levels or treated with insulin-resistant agents like TNF-α to simulate diabetic conditions, GPS has shown notable efficacy. Specifically, GPS promotes the phosphorylation of IRS-1 and Akt, which are critical elements in the insulin signaling pathway. Researchers propose that GPS may influence upstream kinases or phosphatases within this pathway. For instance, it could regulate the activity of protein kinase C (PKC), which phosphorylates IRS-1 at serine residues, thereby inhibiting insulin signaling. By modulating PKC activity, GPS may facilitate the correct phosphorylation of IRS-1 at tyrosine residues, subsequently activating Akt. Once activated, Akt phosphorylates downstream targets, leading to enhanced translocation of GLUT4 to the cell membrane. This process, supported by the upregulation of GLUT4 expression and its movement to the cell surface, ultimately improves cellular glucose uptake.

GPS has the potential to influence the secretion of adipokines, including adiponectin and resistin, which are critical regulators of insulin sensitivity. Adiponectin, a protein produced by adipose tissue, enhances insulin sensitivity through its action on various tissues, such as muscle and liver. In muscle cells, adiponectin stimulates the AMP-activated protein kinase (AMPK) pathway, a central regulator of energy metabolism. Activation of AMPK promotes fatty acid oxidation and glucose uptake (Walte et al., 2024). In the liver, adiponectin inhibits gluconeogenesis, thereby decreasing glucose production. Conversely, resistin, an adipokine associated with insulin resistance and type 2 diabetes, can disrupt insulin signaling. Elevated resistin levels activate the c-Jun N-terminal kinase (JNK) pathway, which phosphorylates IRS-1 at serine residues, impairing its function and resulting in reduced glucose uptake and heightened insulin resistance (Kang et al., 2011).

In a recent investigation involving insulin-resistant rats subjected to a high-fat diet (Wang X. et al., 2023), the administration of GPS was observed to markedly elevate adiponectin concentrations while reducing resistin levels. This biochemical shift was associated with enhanced glucose uptake in muscle cells mediated by insulin. The rise in adiponectin activated the AMPK signaling pathway within muscle tissue, facilitating both fatty acid oxidation and glucose utilization. Simultaneously, the decline in resistin levels mitigated the suppression of insulin signaling, thereby optimizing glucose absorption. These alterations in adipokine expression induced by GPS collectively enhance insulin sensitivity and glucose metabolism. Furthermore, adiponectin exhibits anti-inflammatory characteristics, and its elevation through GPS administration may also attenuate the chronic low-grade inflammation commonly observed in insulin resistance. Conversely, resistin is implicated in inflammatory processes and endothelial dysfunction. The reduction in resistin levels achieved by GPS could potentially ameliorate the metabolic and vascular conditions in individuals with insulin resistance.

#### 4.5.2 Suppression of α-Glucosidase function

α-Glucosidase, an enzyme situated in the brush border of the small intestine, plays a pivotal role in breaking down complex carbohydrates like starches and oligosaccharides into simpler monosaccharides, primarily glucose. This enzymatic activity is essential for the efficient digestion and absorption of dietary carbohydrates. In individuals without metabolic disorders, the function of α-Glucosidase is tightly controlled to facilitate a steady and gradual release of glucose into the bloodstream following meals. However, in cases of diabetes, particularly type 2 diabetes, the unregulated or excessive activity of this enzyme can result in a sharp and uncontrolled rise in postprandial blood glucose levels. Such post-meal hyperglycemia poses significant health risks, as it can lead to chronic damage to vital organs and tissues, including the eyes, kidneys, nerves, and blood vessels (Xiao et al., 2022).

Research has demonstrated that GPS effectively suppresses the activity of α-Glucosidase in laboratory settings (Antoniadi et al., 2023). The proposed mechanism of inhibition involves the targeted interaction of GPS with the enzyme’s active site. The distinct chemical composition of GPS, characterized by its numerous hydroxyl groups and secoiridoid structure, facilitates the formation of non-covalent interactions, including hydrogen bonds, with the amino acid residues within the active site of α-Glucosidase. This interaction interferes with the enzyme’s catalytic efficiency, hindering its ability to break down complex carbohydrates. For instance, *in vitro* experiments involving starch solutions incubated with α-Glucosidase and varying concentrations of GPS revealed a dose-dependent reduction in glucose release, confirming the inhibitory effect of GPS on the enzyme’s functionality.

The suppression of α-Glucosidase activity by GPS plays a pivotal role in decelerating carbohydrate digestion and absorption, which is essential for mitigating the postprandial glucose surge commonly observed in diabetic patients. By slowing down carbohydrate breakdown, glucose enters the bloodstream at a steadier pace, thereby avoiding abrupt spikes in blood sugar levels. Beyond its direct inhibitory effect on α-Glucosidase, GPS may also modulate broader aspects of carbohydrate metabolism. This includes potential regulation of other enzymes or transporters in the small intestine, such as glycosidases or glucose transporters, which are integral to carbohydrate processing and uptake. Such multifaceted mechanisms enhance GPS’s potential as an antidiabetic agent. Furthermore, GPS’s ability to inhibit α-Glucosidase reduces the post-meal insulin requirement. With slower glucose absorption, the pancreas is relieved from excessive insulin secretion, which is particularly advantageous for diabetic individuals with compromised pancreatic function.

### 4.6 Antitumor effects

#### 4.6.1 Induction of tumor cell apoptosis

The antitumor properties of GPS have been extensively studied across multiple cancer cell lines, such as those derived from human lung, breast, and liver cancers. Apoptosis, a form of programmed cell death, is a critical biological process that removes damaged, infected, or abnormal cells. In cancerous conditions, tumor cells frequently acquire the ability to bypass apoptosis, enabling their unchecked growth and metastasis. Research by Liu et al. (2020) demonstrates that GPS treatment effectively triggers apoptosis in these malignant cells (Liu et al., 2020). This is achieved through the activation of the caspase-dependent apoptotic pathway, a primary mechanism responsible for cell death in mammalian systems.

GPS has the potential to enhance the expression of pro-apoptotic proteins, including Bax, which is a critical metabolite of the Bcl-2 protein family. Bax is instrumental in initiating apoptosis by undergoing a structural transformation and migrating to the outer mitochondrial membrane. Once there, it creates pores, facilitating the release of cytochrome c into the cytoplasm. This cytochrome c interacts with Apaf-1, forming the apoptosome complex. This complex then recruits and activates procaspase-9, which subsequently triggers downstream caspases like caspase-3. Caspase-3, as a principal executioner caspase, cleaves multiple cellular substrates, resulting in hallmark apoptotic changes such as DNA fragmentation, chromatin condensation, and cellular shrinkage.

Simultaneously, GPS exerts a down-regulatory effect on the expression of anti-apoptotic proteins, notably Bcl-2. As a critical apoptosis inhibitor, Bcl-2’s overexpression in tumor cells frequently enhances their resistance to programmed cell death. By diminishing Bcl-2 levels, GPS shifts the equilibrium between pro-apoptotic and anti-apoptotic proteins towards apoptosis. This shift triggers the activation of caspases, including caspase-3, -8, and -9. Caspase-8 is primarily activated by cell surface death receptors, such as Fas or TRAIL receptors, and its activation can further propagate the extrinsic apoptotic pathway, leading to caspase-3 activation. The activation of these caspases culminates in the cleavage of cellular substrates, inducing tumor cell apoptosis. For instance, in a study involving human breast cancer cells treated with GPS, researchers observed an elevated Bax/Bcl-2 ratio, caspase activation, and a marked increase in apoptotic cells (Li et al., 2019). These findings underscore GPS’s ability to effectively disrupt tumor cells’ anti-apoptotic mechanisms and induce programmed cell death. Furthermore, GPS may interact with other apoptosis-regulating signaling pathways, such as the PI3K/Akt pathway, which is pivotal in cell survival. By inhibiting the PI3K/Akt pathway, GPS can amplify its apoptotic effects, as activated Akt typically promotes cell survival by phosphorylating and inactivating pro-apoptotic proteins.

#### 4.6.2 Inhibition of tumor cell proliferation and migration

Beyond its role in apoptosis induction, GPS exhibits significant inhibitory effects on tumor cell proliferation and migration, both of which are critical mechanisms driving cancer progression. Experimental studies using *in vitro* cell cultures have demonstrated that GPS treatment effectively halts tumor cells in the G0/G1 phase of the cell cycle (Huang et al., 2020). The cell cycle, a tightly controlled biological process, encompasses multiple phases: G1 (gap 1), S (synthesis), G2 (gap 2), and M (mitosis). In the G0/G1 phase, cells either remain in a dormant state (G0) or prepare for DNA replication in the S phase. GPS disrupts this progression by blocking tumor cells from transitioning into the S phase, thereby preventing DNA replication and subsequent cell division.

The impact of GPS on cellular processes extends to the modulation of proteins critical for cell cycle regulation, including cyclin-dependent kinases (CDKs) and cyclins. CDKs function as enzymes that drive the cell cycle forward by phosphorylating specific target proteins, while cyclins act as regulatory partners that bind to and activate CDKs at distinct phases of the cycle. For instance, the cyclin D-CDK4/6 complex plays a pivotal role in advancing cells through the G1 phase. GPS has been observed to suppress the expression or activity of certain CDKs and cyclins essential for the transition from the G0/G1 phase to the S phase (Li et al., 2019). This suppression may occur through mechanisms such as interference with cyclin D synthesis or stability, or inhibition of CDK4/6 kinase activity. Consequently, GPS effectively halts the cell cycle progression of tumor cells, curbing their uncontrolled proliferation. This mechanism holds significant therapeutic potential, as it can impede tumor growth and offer a promising strategy for cancer treatment.

In the context of cellular migration, GPS has been shown to suppress the activity of matrix metalloproteinases (MMPs), a group of enzymes crucial for the breakdown of the extracellular matrix (ECM). The ECM serves as a structural foundation for cells and tissues, and in cancerous conditions, tumor cells release MMPs to degrade the ECM, facilitating tissue invasion and metastasis. GPS effectively diminishes the expression of specific MMPs, including MMP-2 and MMP-9, at both transcriptional and translational levels. For example, research involving human lung cancer cells demonstrated that GPS treatment significantly lowered the mRNA levels of MMP-2 and MMP-9 (Chen C. et al., 2018). This suppression of MMP expression is mediated through the inhibition of key signaling pathways, such as the mitogen-activated protein kinase (MAPK) pathway, which regulates MMP production. By blocking MAPK activation, GPS curtails MMP expression, thereby preventing ECM degradation and hindering tumor cell migration. The inability to degrade the ECM reduces the likelihood of tumor cells invading adjacent tissues or spreading to distant sites. This anti-migratory property of GPS is a critical component of its potential antitumor effects, as it helps to curb metastasis, a leading cause of cancer-related mortality. Furthermore, GPS may also influence other factors associated with cell migration, such as cell-cell adhesion molecules. By altering the function of these molecules, GPS further disrupts the migratory capacity of tumor cells, offering a multifaceted strategy to inhibit tumor dissemination.

### 4.7 Effects on skin diseases

#### 4.7.1 Psoriasis

Psoriasis, a chronic inflammatory skin condition, impacts millions globally. This disorder is marked by excessive keratinocyte growth and immune cell accumulation, resulting in scaly, itchy skin patches. Research indicates that Gentiopicroside (GPS) holds therapeutic potential for psoriasis. In a study using imiquimod (IMQ)-induced psoriasis in mice, GPS treatment demonstrated significant improvement in skin lesions (Ren et al., 2025; Zhao et al., 2023).

GPS has been shown to modulate the activity of keratinocytes, particularly in the context of psoriasis. In laboratory studies using HaCaT cells—a common model for investigating keratinocyte activation in psoriasis—GPS effectively suppresses the release of inflammatory cytokines and chemokines when cells are exposed to TNF-α and IFN-γ. These cytokines are central to the development of psoriasis, as they trigger keratinocytes to generate inflammatory mediators like IL-8, IL-17, and TWEAK, which are critical for recruiting immune cells to the skin and exacerbating inflammation. By reducing the levels of these mediators, GPS helps mitigate the inflammatory cascade. Furthermore, GPS addresses the excessive production of keratin proteins, such as keratin 16 and keratin 17, which are overexpressed in psoriasis and contribute to skin thickening. GPS normalizes keratinocyte proliferation by downregulating these proteins, thereby restoring a more balanced cellular process.

**FIGURE 3:**
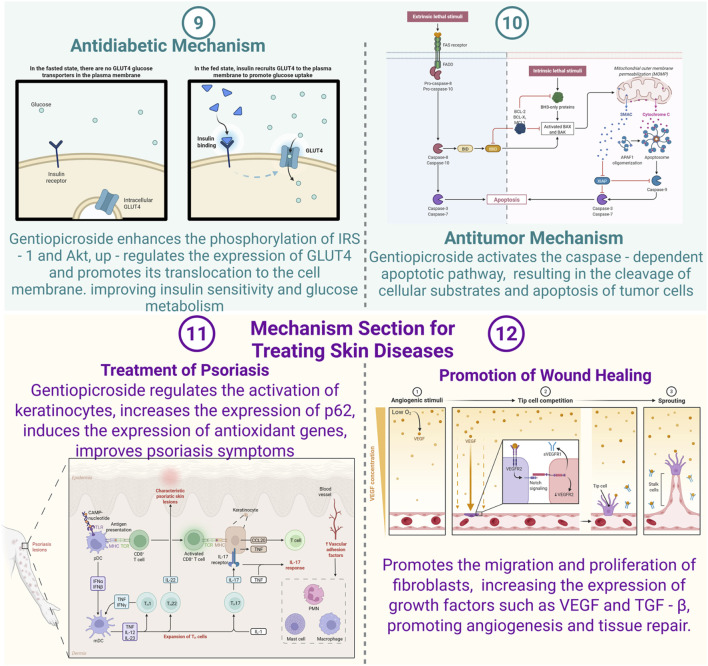
Mechanisms of Gentiopicroside in diabetic, tumor and skin diseases (Created with Biorender.com).

Figure 3 illustrates the regulatory mechanism of the Keap1-Nrf2 signaling pathway. GPS enhances the expression of p62, an adaptor protein that interacts with Keap1, facilitating the release of Nrf2 from Keap1. Once liberated, Nrf2 migrates to the nucleus and binds to antioxidant response elements (AREs) located in the promoter regions of antioxidant genes (Li et al., 2025; Ren et al., 2025; Wan et al., 2021). This interaction triggers the transcription of antioxidant genes, including heme oxygenase-1 (HO-1) and NAD(P)H:quinone oxidoreductase 1 (NQO1). These enzymes mitigate oxidative stress, a critical factor in psoriasis pathogenesis, which drives inflammation and aberrant keratinocyte proliferation. By alleviating oxidative stress, GPS effectively suppresses keratinocyte activation and subsequent inflammatory responses in the skin.

Beyond its impact on the Keap1-Nrf2 signaling axis, GPS exhibits potential regulatory effects on other molecular pathways implicated in psoriasis pathogenesis. Notably, the NF-κB cascade, which is markedly upregulated in psoriatic conditions, serves as a critical mediator for the overexpression of various inflammatory cytokines. GPS may exert inhibitory effects on NF-κB activation through multiple mechanisms, including direct interference or indirect modulation of IκBα phosphorylation and subsequent degradation, mirroring its anti-inflammatory actions in immune cells. This suppression of the NF-κB pathway contributes to the downregulation of pro-inflammatory mediators, thereby enhancing its therapeutic efficacy in psoriasis management. Furthermore, GPS may interact with the JAK-STAT signaling network, a pathway activated by cytokines like TNF-α and IFN-γ in psoriatic lesions. Through its modulation of this pathway, GPS could potentially influence the transcriptional regulation of genes associated with keratinocyte proliferation and differentiation, facilitating the restoration of epidermal homeostasis and skin barrier function.

#### 4.7.2 Wound healing

Research has demonstrated that GPS plays a significant role in enhancing the wound healing process, a multifaceted biological mechanism involving various cellular and molecular interactions. In experimental studies using animal models, such as mice or rats with excisional skin wounds, the application of GPS has been shown to expedite the closure of wounds. Huang et al. (2025) developed a glucose-responsive gel that enhances photodynamic therapy for diabetic abscesses by generating oxygen via glucose oxidase/catalase, while borneol relieves pain and boosts antibacterial efficacy, representing a novel multi-functional approach to wound healing (Huang et al., 2025). This effect has been supported by multiple studies, including those by Li et al. (2024), Zhang Q. L. et al. (2022), Wang et al. (2025), Antoniadi et al. (2023), and Jiang et al. (2021), which collectively highlight the therapeutic potential of GPS in promoting tissue repair and regeneration.

GPS has been shown to significantly enhance the migration and proliferation of fibroblasts, which are essential for collagen synthesis and tissue repair. These cells are crucial in the development of the extracellular matrix during the wound healing process, as they synthesize collagen, providing structural integrity and strength to the regenerating tissue. The mechanism by which GPS influences fibroblasts involves multiple signaling pathways. For instance, GPS activates the PI3K/Akt pathway, leading to increased phosphorylation of Akt, which enhances cell survival, proliferation, and migration (Liu et al., 2012; Wang X. et al., 2023; Xiao et al., 2022; Xing et al., 2021; Yin et al., 2024). This activation results in fibroblasts exhibiting greater motility and higher proliferation rates, accelerating the closure of wounds. Additionally, GPS upregulates the expression of focal adhesion proteins like vinculin and talin, which are critical for cell-matrix adhesion and cellular movement (Wang et al., 2025; Wang X. et al., 2023; Xiao et al., 2022; Xing et al., 2021; Yin et al., 2024). By boosting the expression of these proteins, GPS improves the fibroblasts’ ability to migrate to the wound site, thereby facilitating the repair process.

As illustrated in Figure 3, GPS has been shown to elevate the levels of key growth factors, including vascular endothelial growth factor (VEGF) and transforming growth factor-beta (TGF-β). VEGF plays a pivotal role in angiogenesis, the formation of new blood vessels, which is critical for delivering oxygen and nutrients to healing tissues while eliminating metabolic waste. TGF-β, on the other hand, contributes to various stages of wound healing, such as fibroblast activation, collagen production, and tissue regeneration. The upregulation of these growth factors by GPS enhances both angiogenesis and tissue repair processes. For example, in a murine wound model, GPS treatment resulted in a marked increase in VEGF expression within the wound site, correlating with a higher density of blood vessels (Berthon et al., 2023; Wang et al., 2025; Yang et al., 2018; Zhang L. et al., 2023). This improved vascularization creates a more favorable environment for the migration and proliferation of fibroblasts and other cell types essential for wound healing. Additionally, TGF-β promotes collagen synthesis by fibroblasts, further supporting tissue remodeling and recovery.

The extracellular matrix metabolites play a critical role in wound contraction and tissue regeneration, as highlighted in several studies (Almukainzi et al., 2022b; Avila-Carrasco et al., 2019; Chen C. et al., 2024; Jiang et al., 2021; Zhao et al., 2015). GPS, with its anti-inflammatory and antioxidant properties, contributes to creating an optimal microenvironment for wound recovery. While inflammation is a natural response to injury, excessive or prolonged inflammation can hinder the healing process. GPS mitigates this by suppressing the release of pro-inflammatory cytokines like TNF-α and IL-6 at the wound site (Chang et al., 2021; Hu et al., 2024; Konya et al., 2023; Wang et al., 2020; Xie et al., 2019). This reduction in inflammation not only prevents further tissue damage but also accelerates the transition from the inflammatory phase to the proliferative phase. Additionally, GPS’s antioxidant capabilities counteract oxidative stress, a byproduct of reactive oxygen species (ROS) generated during wound healing. Elevated ROS levels can harm cellular structures such as DNA, proteins, and lipids, impairing the function of cells essential for repair. By neutralizing ROS, GPS supports the viability and activity of fibroblasts, endothelial cells, and other cell types crucial for tissue regeneration (Chen C. et al., 2024; Jin et al., 2020; Li et al., 2025; Yin et al., 2024; Zhang L. et al., 2023).

### 4.8 Hepatoprotective effects

#### 4.8.1 Protection against chemical - induced liver injury

Liver damage caused by chemical substances, such as pharmaceuticals, alcohol, and environmental pollutants, is a prevalent issue. Research has demonstrated that GPS exhibits hepatoprotective properties in mitigating such injuries. Specifically, in experimental models utilizing carbon tetrachloride (CCl_4_), a recognized hepatotoxic agent, GPS administration has been shown to markedly alleviate hepatic damage (Mihailović et al., 2014; Zhang Y. et al., 2023).

The metabolism of CCl_4_ in the liver involves cytochrome P450 enzymes, which generate trichloromethyl radicals. These radicals interact with lipids in cell membranes, initiating lipid peroxidation. This process damages cell membranes, impairs cellular functions, and results in cell death. GPS has been shown to mitigate lipid peroxidation by enhancing the activity of key antioxidant enzymes, including superoxide dismutase (SOD), catalase (CAT), and glutathione peroxidase (GSH-Px). These enzymes are essential for neutralizing reactive oxygen species (ROS) and safeguarding cells from oxidative stress. Specifically, SOD facilitates the conversion of superoxide anions into hydrogen peroxide, which is subsequently broken down by CAT and GSH-Px. In a study involving a CCl_4_-induced liver injury model, GPS administration was associated with elevated activities of SOD, CAT, and GSH-Px in liver tissue, alongside reduced levels of malondialdehyde (MDA), a lipid peroxidation marker (Mihailović et al., 2014; Zhang Y. et al., 2023).

The liver’s inflammatory cytokine expression can be modulated by GPS. When the liver is subjected to CCl_4_-induced damage, it releases cytokines like TNF-α, IL-1β, and IL-6, which are known to promote inflammation. These cytokines attract immune cells to the liver, intensifying the inflammatory response and causing additional liver injury. GPS has the ability to suppress the expression of these pro-inflammatory cytokines. It likely exerts its effects by targeting the NF-κB pathway, a crucial regulator of cytokine production. By blocking NF-κB activation, GPS decreases the transcription of genes responsible for producing pro-inflammatory cytokines. This mechanism helps mitigate liver inflammation and shields liver cells from further harm.

Beyond its antioxidant and anti-inflammatory properties, GPS has been shown to facilitate the regeneration of liver cells. This is evidenced by its ability to elevate the levels of proliferating cell nuclear antigen (PCNA), a key indicator of cellular proliferation. In studies involving a CCl_4_-induced liver injury model, treatment with GPS resulted in a higher count of PCNA-positive cells within the liver, suggesting a boost in liver cell regeneration. Furthermore, GPS appears to positively influence the activation of hepatic stellate cells (HSCs). Typically dormant under normal conditions, HSCs become active during liver damage, transforming into myofibroblasts that contribute to liver fibrosis. GPS, however, seems to regulate HSC activation in a manner that encourages the secretion of growth factors and cytokines conducive to liver healing, thereby preventing excessive fibrosis.

#### 4.8.2 Mitigation of liver damage caused by alcohol

Liver injury caused by alcohol consumption represents a significant global health concern, with its progression ranging from initial fatty liver to advanced stages like alcoholic hepatitis and liver cirrhosis. Research conducted on animal models has demonstrated that GPS can effectively mitigate the detrimental effects of alcohol on liver health (Yang H. X. et al., 2020; Zhang et al., 2021).

The primary sites for alcohol metabolism are the liver, where enzymes such as alcohol dehydrogenase (ADH) and cytochrome P450 2E1 (CYP2E1) play crucial roles. During this process, acetaldehyde, a harmful byproduct, and reactive oxygen species (ROS) are produced. Acetaldehyde has the ability to bind with proteins, creating adducts that impair liver cell integrity and disrupt cellular functions. Simultaneously, ROS induce oxidative stress, which can lead to lipid peroxidation, protein oxidation, and DNA damage. GPS has been shown to boost the activity of both ADH and aldehyde dehydrogenase (ALDH), the latter being essential for converting acetaldehyde into acetate. By enhancing the efficiency of these enzymes, GPS facilitates the faster breakdown of alcohol and acetaldehyde, thereby mitigating their detrimental impact on liver health.

GPS has been shown to mitigate oxidative stress in liver tissues affected by alcohol-induced damage. By enhancing the activity of key antioxidant enzymes, including SOD, CAT, and GSH-Px, GPS exerts a protective effect similar to its role in chemical-induced liver injury. In experimental models of alcohol-induced liver damage, GPS administration significantly elevated the levels of these enzymes while reducing the concentrations of reactive oxygen species (ROS) and malondialdehyde (MDA). This antioxidative mechanism plays a crucial role in shielding liver cells from oxidative harm and preserving their functional integrity.

Additionally, GPS has been shown to influence hepatic lipid metabolism. Excessive alcohol intake frequently causes fat to build up in the liver, leading to steatosis. GPS has the ability to control the expression of genes that are involved in lipid metabolism, including peroxisome proliferator-activated receptor-α (PPAR-α) and sterol regulatory element-binding protein-1c (SREBP-1c). PPAR-α is a transcription factor that controls the oxidation of fatty acids, whereas SREBP-1c plays a role in the synthesis of fatty acids. GPS can increase the expression of PPAR-α and decrease the expression of SREBP-1c, thereby enhancing fatty acid oxidation and reducing fatty acid synthesis in the liver. This mechanism helps to decrease lipid accumulation in the liver and prevent the onset of steatosis.

## 5 Pharmacokinetics

Research conducted on animal models has explored the gastrointestinal absorption of GPS. Following oral intake, GPS demonstrates partial absorption, yet its bioavailability remains limited. This limitation is attributed to issues such as low solubility in the gastrointestinal environment and significant first-pass metabolism (Chang-Liao et al., 2012; Fu et al., 2023; Tong et al., 2023; Wang et al., 2007; Wang et al., 2004). To address these challenges, studies have investigated strategies like co-administering absorption enhancers or modifying formulations. For instance, encapsulating GPS within nanoparticles or liposomes has been shown to improve its solubility and shield it from gastrointestinal degradation, thereby potentially enhancing absorption (Almukainzi et al., 2022a; Almukainzi et al., 2022b; Zhang et al., 2013). High-sensitivity techniques, such as Guo et al.’s (2024) UV/Vis colorimetric method for triterpenes and Peng et al.’s (2024) UPLC-QTOF-MS-based profiling of TCM constituents, address key analytical challenges in pharmacokinetics and formulation, offering tools applicable to GPS research for metabolite quantification and characterization (Guo et al., 2024; Peng et al., 2024).

After absorption, GPS is transported to multiple tissues throughout the body. Research utilizing radiolabeled GPS has demonstrated its presence in organs including the liver, kidneys, brain, and lungs (Li et al., 2012; Sun et al., 2022; Wang et al., 2004). Within the liver, GPS undergoes further metabolic processes, while in the brain, it contributes to neuroprotective activities. The tissue-specific distribution of GPS is closely linked to its therapeutic roles in addressing diseases associated with these organs. Nevertheless, the precise mechanisms behind its targeted distribution, particularly the involvement of transporters, remain incompletely elucidated (Ding et al., 2024; Shi et al., 2020; Ye et al., 2023).

The primary site of GPS metabolism is the liver, where cytochrome P450 enzymes play a significant role in its biotransformation, resulting in the generation of diverse metabolites (Almukainzi et al., 2022b; Chang-Liao et al., 2012; Dai et al., 2018; Deng et al., 2013). These metabolites may exhibit pharmacological effects, such as antioxidant or anti-inflammatory properties. Investigating the metabolic pathways of GPS is crucial for understanding its pharmacokinetic behavior and anticipating possible drug-drug interactions, particularly since substances that modulate cytochrome P450 enzyme activity can alter GPS metabolism (Chang-Liao et al., 2012; Deng et al., 2013; Liu et al., 2024).

## 6 Toxicity and safety

Research into the acute toxicity of GPS in animal models, including mice and rats, indicates that GPS exhibits a relatively low level of acute toxicity. While high doses administered over a short duration can induce symptoms such as lethargy, decreased food consumption, and weight loss in these animals, no significant mortality has been observed within specific dosage limits (Ding et al., 2024; El Gizawy et al., 2019; Mihailović et al., 2013). Nonetheless, the median lethal dose (LD50) can differ based on the species of animal and the method of administration (El Gizawy et al., 2019; Feng et al., 2014; Wang et al., 2004).

In studies examining sub-chronic and chronic toxicity, animals are subjected to prolonged exposure to GPS. Research indicates that extended administration of GPS at suitable dosages typically does not result in substantial harm to vital organs. Nonetheless, when administered at excessively high concentrations, alterations in hepatic and renal functionality may occur. For instance, there could be a marginal elevation in serum ALT and AST levels, alongside modifications in the histological architecture of the kidneys (Cao et al., 2025; Xu et al., 2022; Zhang Y. et al., 2023). These observations underscore the necessity for a thorough assessment of GPS safety, particularly in the context of its prolonged clinical application (Bampidis et al., 2023; Ding et al., 2024; Liu et al., 2024).

In terms of genotoxicity, comprehensive evaluations using both *in vitro* and *in vivo* methodologies have been conducted. The Ames test, a widely recognized *in vitro* assay, demonstrated that GPS does not induce mutations in bacterial strains. Similarly, the *in vivo* micronucleus test revealed no significant increase in the frequency of micronucleated erythrocytes in the bone marrow of mice (Bampidis et al., 2023; Cvetković et al., 2020; Mustafayeva et al., 2010). While current evidence does not suggest that GPS possesses carcinogenic properties, it is imperative to conduct long-term carcinogenicity studies to thoroughly evaluate its safety profile in this context (Bampidis et al., 2023; Ding et al., 2024; Liu et al., 2024).

## 7 Clinical studies and prospects

While direct clinical trials focusing on the application of GPS in human diseases remain scarce, research into traditional medicinal formulations incorporating GPS-rich plants has gained traction, particularly in the context of liver disorders and inflammatory conditions. For instance, clinical studies involving patients with chronic hepatitis have explored the efficacy of botanical drug preparations containing Gentiana scabra Bunge, a plant abundant in GPS, in enhancing liver function and alleviating symptoms (Huang et al., 2024; Yang H. X. et al., 2020; Yetmar et al., 2023). These investigations offer indirect insights into the possible therapeutic benefits of GPS in clinical settings (Ding et al., 2024; Liu et al., 2024; Wang et al., 2015).

Although this study has systematically elucidated the therapeutic efficacy and mechanisms of GPS in various diseases, its clinical translation still faces significant challenges. Future research should focus on conducting more detailed preclinical studies to clarify its exact molecular mechanisms in various diseases, particularly in complex conditions like neurodegenerative disorders (e.g., Parkinson’s disease) and cancer (Gao et al., 2025; Xiong et al., 2024; Yin et al., 2024). Efforts also should be directed toward developing formulations that enhance the bioavailability of GPS(Almukainzi et al., 2022a; Mudrić et al., 2025; Tong et al., 2023). The oral bioavailability of GPS is relatively low (approximately 12%–18%), primarily due to its poor water solubility caused by multiple polar hydroxyl groups in its molecular structure (Chang-Liao et al., 2012; Ren et al., 2023). Furthermore, existing studies are mostly based on *in vitro* cell models and animal experiments, which can only preliminarily verify the safety of GPS; large-scale clinical data supporting its efficacy and long-term safety are lacking. Therefore, this study suggests that these limitations can be overcome from the following aspects.

Firstly, it is recommended to adopt nanocrystal technology (e.g., aprepitant nanocrystals) or self-assembled prodrug nanoparticles (e.g., darutigenol prodrug nanoparticles) to improve the water solubility and intestinal absorption efficiency of GPS. Prof. Zhang from Peking University have shown that when the particle size of nanocrystals is reduced from 2.5 μm to 200 nm, the bioavailability can be enhanced by 3-5 fold (Almukainzi et al., 2022b). The ROS-responsive prodrug nanoparticles developed by Prof. Jia’s team have demonstrated targeted accumulation capability in arthritis models, which can reduce systemic toxicity and enhance therapeutic efficacy (Wang et al., 2020). Secondly, it is advisable to conduct multicenter, randomized controlled phase II clinical trials, focusing on evaluating the synergistic effects of GPS combined with chemotherapy (e.g., cisplatin). Metabolomics and digital twin technology should be integrated to predict individual pharmacokinetic differences, thereby achieving precise drug administration. Thirdly, clinical trials need to be meticulously designed to assess the efficacy and safety of GPS in targeted diseases, such as randomized, double-blind, placebo-controlled studies in patients with psoriasis or neurodegenerative conditions (Antoniadi et al., 2023; Ding et al., 2024; Liu et al., 2024).

Fourthly, regulatory considerations and quality control must be strengthened. GPS content varies significantly among plant species, origins, and extraction methods (Li et al., 2020; Zhou et al., 2010). Standardizing raw material sourcing, extraction processes, and formulation quality is critical to ensuring batch consistency and reproducible therapeutic effects. Regulatory agencies should establish guidelines for GPS-based pharmaceuticals, including specifications for purity, stability, and impurity limits (EFSA Panel on Additives and Products or Substances used in Animal Feed, 2023; Chinese Pharmacopoeia Commission, 2020). Additionally, long-term toxicity studies are needed to evaluate potential hepatotoxicity or nephrotoxicity, as high-dose Pb exposure has been shown to attenuate GPS’s hepatoprotective effects in animal models (Zhang R. et al., 2023) A standard for monitoring GPS plasma concentrations should be established, and LC-MS/MS should be used to determine plasma drug concentrations to ensure detection sensitivity and precision. Finally, exploring combination therapies could amplify therapeutic outcomes while mitigating adverse effects. For example, combining GPS with nonsteroidal anti-inflammatory drugs (NSAIDs) may reduce NSAID-induced gastrointestinal toxicity through GPS’s anti-inflammatory and gastroprotective effects (Yang et al., 2018). In cancer treatment, integrating GPS with chemotherapy or immunotherapy may enhance cytotoxicity against tumor cells (e.g., by inhibiting the PI3K/AKT pathway in gastric cancer) while reducing harm to healthy tissues (Chen Q. et al., 2024; Lu et al., 2024). Integrating GPS with conventional anti-cancer treatments may enhance cytotoxicity against tumor cells while reducing harm to healthy tissues (Chen Q. et al., 2024; Lu et al., 2024; Yin et al., 2024).

## 8 Conclusion

GPS demonstrates significant therapeutic potential across a spectrum of diseases, showcasing anti-inflammatory, antioxidant, neuroprotective, hepatoprotective, antidiabetic, antitumor, and skin-related therapeutic properties. Its mechanisms of action are attributed to the modulation of diverse signaling pathways and cellular functions. Despite the need for further enhancement and assessment of its pharmacokinetics and safety profile, GPS emerges as a promising lead metabolite for novel drug development. Future research should prioritize the translation of pre-clinical discoveries into clinical practice via meticulously designed trials and the refinement of its pharmacological characteristics.

While the current research on GPS shows significant promise, it is crucial to acknowledge that additional investigations are necessary to comprehensively evaluate its therapeutic potential and inherent limitations. The existing literature provides a robust basis for further exploration of this metabolite’s applications in medicine. As the field advances, it is anticipated that new discoveries will enhance our understanding, paving the way for more effective and targeted use of GPS in clinical settings.
